# Quality Factors of Commercial Snail Fillets as Affected by Species

**DOI:** 10.17113/ftb.60.03.22.7403

**Published:** 2022-09

**Authors:** Efkarpia Kougiagka, Chrysoula Apostologamvrou, Persephoni Giannouli, Marianthi Hatziioannou

**Affiliations:** 1 Department of Ichthyology and Aquatic Environment, School of Agricultural Sciences, University of Thessaly, Fytoko Street, 38 446, Nea Ionia Magnesia, Greece; 2 Department of Biochemistry and Biotechnology, School of Health Sciences, University of Thessaly, Viopolis, 41500, Larissa, Greece

**Keywords:** snail fillets, hardness evaluation, proximate composition, histological structure, colour measurement

## Abstract

**Research background:**

This study fulfils a need for investigation of a quality profile of snail fillets. Edible snails are a famous food product consumed worldwide and treated as delicacy. Nutritional value, colour and textural properties, such as hardness, are critical factors that impact consumer acceptance of the product. Hardness of snail meat is affected by its native original microstructure.

**Experimental approach:**

Fresh snails of the farmed species *Cornu aspersum maximum,* wild and farmed *Cornu aspersum aspersum* and wild *Helix lucorum* were used in order to investigate the qualitative profile of snail meat. Proximate composition, hardness and colour measurements were conducted on fillets of all species. The histological structure of the fillets of *Cornu aspersum maximum* was examined.

**Results and conclusions:**

Quality parameters of snail fillets were studied. A novel method of hardness analysis was proposed where the cylindrical part of snail fillets from the mid-posterior region with specific geometry 6 mm diameter and 6 mm height was used. The suitability of the mid-posterior region was enhanced by the uniform structure confirmed by the histological analysis. *Helix lucorum* snail fillet had the highest energy content and the highest hardness but the lowest carbohydrate content. The species *Cornu aspersum maximum* was evaluated with the highest values of a* (redness), b* (yellowness) and C* (chroma) compared to other species. Parameter L* (lightness) of wild snail fillets was lower than of the farmed ones due to age, diet, farming or environmental conditions, but it could also be related to snail carbohydrate content.

**Novelty and scientific contribution:**

This study yielded notable results on qualitative characteristics of snail fillets as food and important information is given on its meat properties. Furthermore, a novel methodology of hardness is provided in order to minimize natural, breeding and environmental influences. Finally, the research outcomes could lead to proper handling methods for further fabrication of snail meat.

## INTRODUCTION

Invertebrates constitute an important component of the diet worldwide and many molluscan species are known for their culinary value (*1*, *2*). Among gastropods, the helicine species (*Helix pomatia, Helix lucorum, Cornu aspersum*), consumed extensively in Europe, have been the principal subject of studies related to proximate composition (*3*-*5*), while the organoleptic and mechanical properties have been studied to a lesser extent.

Τhere is no terminology about the edible parts of the snail. Namely, the whole snail is usually referred to as snail meat (*3*, *6*) and the foot-head mass which is the main edible part (*7*) is mentioned as foot (*8*, *9*), foot muscle (*7*) and pedal mass (*4*). The body of the edible terrestrial snails is covered with epithelium and is protected by a shell secreted by specialized epithelial cells. Underlying the epithelial layer of the gastropod’s integument, complex arrays of muscle and connective tissue complete its basic structure (*10*). In literature, typical histological structure has been reported for many species (*10*). The subepidermal connective tissue was traversed by different types of cells such as rhogocytes, glycogen cells and secretory cells that contain proteins, calcium, pigments, fat globules and mucus. In the lower layer of subepithelial tissue, between muscle and connective tissue, there are empty spaces, the haemocoelic sinuses where haemolymph is gathered. Foot mucus functional role is affected by proximate composition including 90–99.7% water and a glycoprotein complex (*11*). Greistorfer *et al.* (*11*) reported four types of mucus glands in the foot of the species *C. a. aspersum*. Histology of snail fillet might present differences in the size of muscle cells (*12*) and collagen fibre diameter (*13*) related to species, age, diet and farming conditions. The histological analysis of meat products could also guarantee the authenticity in fraud cases (*14*) and is used for texture optimization after various treatments in order to meet consumer needs (*15*).

Edible snail tissue is a potential source of protein and essential nutrients such as vitamins and minerals, especially calcium, potassium, sodium and trace elements such as iron and selenium (*3*, *4*). Protein and carbohydrates play major roles in providing the desirable rheological and textural attributes in meat and fish (*16*). Hardness is an important factor for the food industry as this texture parameter is related to the way the food is processed and to the acceptance by the consumers. Nowadays, textural parameters have been studied extensively in various types of meat such as beef and lamb and it is well known that breeding, postharvest handling, and product preservation are also parameters that affect hardness of the final meat products (*17*-*19*). Texture profile analysis (TPA) is a popular method for determining textural properties of foods and evaluating quality factors when sensory experiments are expensive, time-consuming and complex as for example for raw meat samples (*20*). In other studies, tissue parts of specific dimensions were used for the texture assessment of invertebrates and vertebrates (*21*). Mizuta *et al.* (*21*) used a part after the head of the prawn in order to estimate muscle firmness, and Cimmino *et al.* (*17*) obtained pieces of goat meat parallel to the longitudinal orientation of the muscle fibres assessing meat tenderness. Schubring and Meyer (*22*) noticed significant differences between the values of hardness and chewiness of the species *C. a. aspersum* and *Achatina fulica.* The authors reported that *C. a. aspersum* had the lowest values and *A. fulica* the highest values of the aforementioned parameters compared with *H. pomatia* and *H. lucorum.* Nevertheless, the above approach mainly focusses on all snail meat properties and particularly on the cooked meat or does not take into account the variability in raw snail fillet quality properties. Texture analysis of the raw meat of snail fillets could give further information on the technical and economic aspects of food processing.

Another important sensory attribute of food is colour, which also affects sales and profitability. Many researches showed that visual selection of snails is based on shell size and shell colouration diversity (*23*, *24*). Also, high frequencies of dark shells have been mentioned in shaded environments and pale ones in hotter areas (*25*, *26*). Except for shell, snail meat colour is also very important. Edible tissue of fresh wild snails *C. a. aspersum* had high values of lightness L* and yellowness b* and low value of redness a* (*3*). Also, processed snail meat colour parameters after deep freezing were important quality characteristics of snails collected in Lithuania (*27*). Investigation of the chemical composition of snail meat and colour is very important to give the magnitude of impact on variations among different snail species.

In our research we evaluate for the first time the quality properties of snail fillets of different commercial species, farmed and wild. The primary aim is to understand the association of histology, chemical analysis, hardness and colour parameters of each kind of four commercial snail fillets, farmed *Cornu aspersum maximum,* farmed and wild *C. a. aspersum* and wild *H. lucorum.* Additionally, a new method for texture analysis is also proposed based on the shape and size of a specific part of snail fillet sample in order to optimize hardness assessment. Elucidating quality factors for commercial snail fillets, we aim to help standardize quality parameters and give further information on the technical aspects of snail food processing.

## MATERIALS AND METHODS

### Snail samples and preparation

**Fig. S1:**
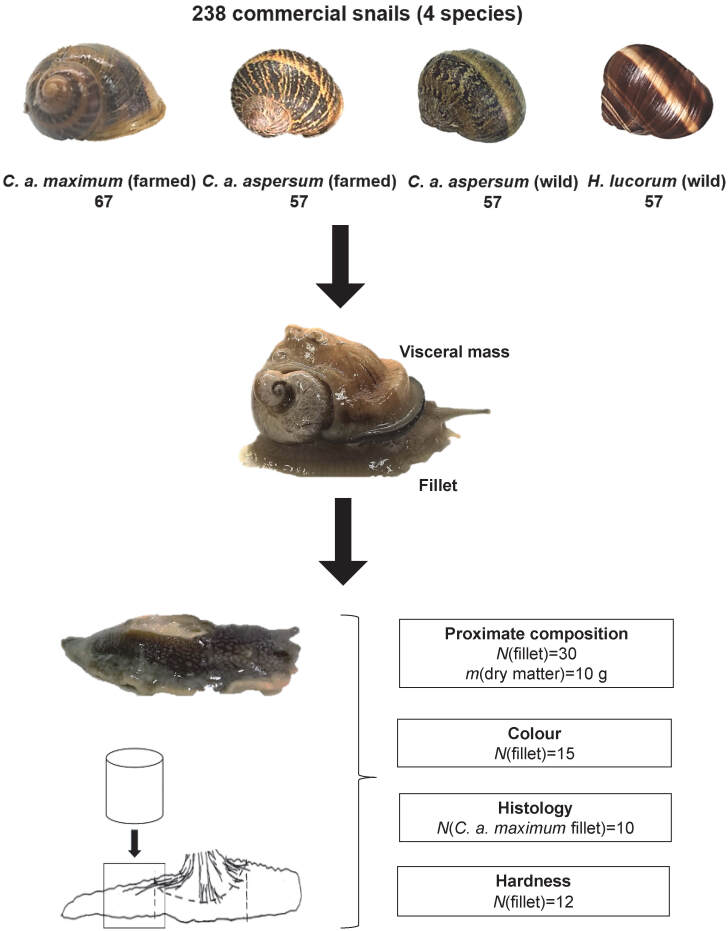
Scheme of snail preparation before analyses In legend write: First one: *N*(fillet)=30 *m*(dry matter)=10 g Second one: Colour *N*(fillet)=15 Third one: *N*(fillet *C. a. maximum*)=10 Fourth one: *N*(fillet)=12

For the experimental procedure, we used market-size snails of four commercial species consumed worldwide: farmed *Cornu aspersum maximum*, farmed *Cornu aspersum aspersum,* wild *C. a. aspersum* and wild *Helix lucorum* (Fig. S1). Farmed *C. a. maximum* snails were supplied from a net covered greenhouse (Volos, Magnesia, Thessaly, Greece) and *C. a. aspersum* from an open field farm (Kontariotissa, Pieria, Central Macedonia, Greece). Wild *C. a. aspersum* snails and wild *H. lucorum* were purchased from local retailers in Heraklion (Crete, Greece) and Serres (Central Macedonia, Greece) respectively. All snails were transported to the laboratory 2 days after their collection, in April 2019.

A total of 238 fresh snails of the four aforementioned species were used for the analyses (Fig. S1). The mass (*m*) of whole raw snails and three morphometrical characteristics of each snail shell were measured in each specimen: shell diameter (*d*), shell height (*h*) and shell aperture diameter (*d*_a_) using a precision balance (EMB 200-2; Kern & Sohn, Balingen, Germany) and a digital caliper (Fowler High Precision, Canton, MA, USA) both with two decimal places. Additionally, the mass of raw fillet (*m*_f_) was recorded after shell and visceral mass removal (Fig. S1) and then histological, compositional, textural and colourimetric analyses were performed.

The histological analysis was conducted only on *C. a. maximum* (10 raw fillets), while the other analyses were performed on all species. Colour was assessed of 15 fillets per species, textural analysis of 12 fillets per species, and 30 fillets per species were used for proximate composition analysis (Fig. S1). Apart from whole fillets, cylindrical parts with 6 mm in diameter and 6 mm height from the mid-posterior region of fillets were used for textural and histological analyses. The entire analysis procedure is shown in Fig. S1.

### Histological analysis

The histological analysis was carried out in 10 snail fillets of farmed *C. a. maximum,* after anaesthesia using twenty drops of *Eugenia caryophyllus* oil (clove oil, CHEMCO, Malsch, Germany) diluted in 50 mL water (*28*). Snails were kept in this emulsion for 2 h at room temperature in order to relax and extend their body.

The specimens of fresh fillets, removed from the mid-posterior region of the fillet (Fig. S1), were fixed in 10% neutral buffered formalin (Epredia™ 10% Neutral Buffered Formalin; Thermo Fisher Scientific, Waltham, MA, USA) and were placed in cassettes and put in histokinette (TP 1020; Leica, Wetzlar, Germany) for dehydration (immersion in ethanol solution of increasing concentrations), then cleared with immersion in xylene solutions (Thermo Scientific, Thermo Fisher Scientific) to replace ethanol with an organic dissolvent and embedded in liquid paraffin wax (Fisherbrand™ Histoplast Paraffin Wax; Thermo Scientific, Thermo Fisher Scientific) using heated paraffin embedding station (EG 1159H; Leica). Paraffin blocks were left to cool (EG 1150C; Leica); then, the mould was removed and the blocks were mounted on a microtome (CUT 5062; SLEE medical GmbH, Mainz, Germany) for cutting (5 µm slices). The slices were stained with the acidified haematoxylin (Thermo Scientific™ Shandon™ Harris hematoxylin; Thermo Fisher Scientific) and alcoholic eosin (eosin Y; Thermo Fisher Scientific) regressive staining procedure, covered with Canada balsam mounting medium, and observed under Carl Zeiss light microscope (Carl Zeiss Ltd, Gottingen, Germany) connected to a ProgRes C10 digital camera (Berlin, Germany), and subsequently processed through image analysis using the software ProgRes Capture Pro 2.1 (Berlin, Germany). Some slices were additionally stained using the Masson’s trichrome staining methods (Masson’s trichrome kit with aniline blue DC; Panreac Química SAU, Barcelona, Spain).

### Proximate composition

Proximate composition of 30 raw fillets per species was assessed according to AOAC (*29*). For moisture mass fraction determination (%), 3 g were dried at 105 °C in an oven (TS 8056; Termaks, Bergen, Norway) until constant mass, and the water content was determined gravimetrically (*29*). Dry matter of fillets of each species was pooled and 10 g of dry matter were used for the following analyses. Crude protein mass fraction (%) was tested by the Kjeldahl method (N×6.25; Behr Labor-Technik, Düsseldorf, Germany) as it is referred in literature about proximate composition of snail meat (*5*) using 0.2 g dry matter and then expressed on net basis and crude fat by Soxhlet method (SOXTHERM® SOX416 macro; Gerhardt, Königswinter, Germany). With regard to ash mass fractions (%), a water-free sample was combusted in a muffle furnace (Nabertherm L9/12/C6; Lilienthal, Germany) by heating at 600 °C for 3 h and the ash content was measured gravimetrically. Gross energy (KJ/g) was evaluated using an adiabatic oxygen bomb calorimeter (C7000; IKA Werke, Staufen, Germany). Crude protein, crude fat and ash mass fractions of each group were expressed on net basis. Carbohydrate mass fraction (%) was calculated by the difference between 100 and the sum of the crude protein, crude fat and ash content on net basis (*30*). All measurements were carried out in triplicate and the values were averaged.

### Hardness measurement

For hardness measurements, 6 whole fresh fillets and 6 cylindrical (*d*=6 mm and *h*=6 mm) parts from the mid-posterior region of fillets of each species (Fig. S1) were analyzed at 5 °C. The instrumental texture was measured using texture analyzer (Admet eXpert 5601; AdMEt, Inc., Norwood, MA, USA) and cylindrical probe of 18 mm diameter. The instrumental texture measurements were performed as texture profile analysis (TPA) at 75% compression and the speed was 100 mm/min. According to Schubring and Μeyer (*22*) and Ruiz De Huidobro *et al.* (*18*), the texture attribute ’hardness’ is defined as the maximum force of the first compression (*F*_max_). For each group of whole fresh snails and cylindrical parts of farmed *C. a. maximum*, farmed and wild *C. a. aspersum* and wild *H. lucorum*, average *F*_max_ values were evaluated.

### Colour measurement

Colour measurements were performed at 5 °C on the surface of the ventral region of fresh *C. a. maximum*, farmed and wild *C. a. aspersum* and *H. lucorum* fillets using colourimeter (HunterLab MiniScan XE Plus, Reston, VA, USA) in the CIELAB colour space (*31*). Lightness (L*), redness (a*) and yellowness (b*) were recorded per each kind of snail fillets (Fig. S1). L* or lightness express dark to light scale of 0 to 100. The parameters a* or redness shows green to red and b* or yellowness represents blue to yellow both on a scale from −60 to +60. Also hue (*h*^0^) or hue angle was determined by the equation:

*h*^0^=arctan(b*/a*) /1/

and chroma (C*) or the colour saturation index was calculated manually according to the AMSA method (*32*):



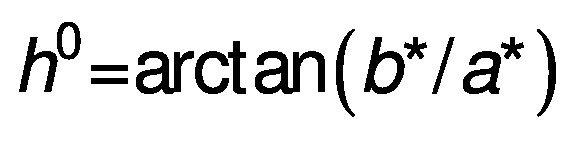



Three replicate measurements were obtained and averaged. The 15 fillets were divided in five groups of three snail fillets.

### Statistical analysis

The data were analyzed using SPSS Statistics 23 (*33*) and expressed as mean value±standard deviation (S.D.). One-way analysis of variance (ANOVA) followed by Tukey’s multiple comparison test at the significant level of 0.05 were used to compare the data of morphometrical characteristics of snail shell and mass of whole snails and snail fillets of different species (*34*).

## RESULTS AND DISCUSSION

### Morphological characteristics of snails

The snail species of this study belonged to the list of farmed and wild edible species used in food manufacture (*5*, *35*, *36*). Differences in size, mass and shell morphometrical characteristics among species are observable (Table 1). These differences were explained by the species, age of snails, period of collection, breeding conditions and diet (*4*, *5*, *37*, *38*).

**Table 1:** Morphological characteristics of the different snail species

Species	*d*/mm	*h*/mm	*d*_a_/mm	*m*/g	*m*_f_/g
*C. a. maximum* (farmed)	(40.0±3.3)**^a^**	(39.2±3.8)**^a^**	(23.1±3.5)**^a^**	(21.3±3.6)**^a^**	(2.1±0.6)**^a^**
*C. a. aspersum* (farmed)	(30.9±1.8)**^b^**	(30.5±2.2)**^b^**	(16.8±1.6)**^b^**	(8.4±1.4)**^b^**	(1.1±0.4)**^b^**
*C. a. aspersum* (wild)	(31.6±2.4)**^b^**	(30.8±2.2)**^b^**	(16.5±1.4)**^b^**	(7.8±1.2)**^b^**	(1.0±0.3)**^b^**
*H. lucorum* (wild)	(35.1±3.3)**^c^**	(31.9±3.2)**^b^**	(16.3±2.9)**^b^**	(12.7±2.5)**^c^**	(1.6±0.6)**^c^**

Results are expressed as mean value±S.D. (*N*=57). Data within the same column marked with different lowercase letters are significantly different at p<0.05 according to ANOVA. *d*=snail shell diameter, *h*=snail shell height, *d*_a_=snail shell aperture diameter, *m*=mass of whole raw snail, *m*_f_*=*mass of raw fillet

As it concerns our samples, shell diameter (*d*), a parameter of snail size, ranged from 30.9 to 40.0 mm. *Cornu aspersum maximum* ((40.0±3.3) mm) and *Helix lucorum* ((35.1±3.3) mm) had significantly higher *d* value from farmed ((30.9±1.8) mm) and wild ((31.6±2.4) mm) *Cornu aspersum aspersum* (p<0.05). Similar statistical differences in *d* were also reported for the mass of snails. More specifically, mass of wild *C. a.aspersum* with (7.8±1.2) g and farmed *C. a. aspersum* with (8.4±1.4) g were significantly (p<0.05) lower than *H. lucorum* (12.7±2.5) g and *C. a. maximum* (21.3±3.6) g snail mass (Table 1).

Even though the fillet is the main edible part of snails and is used in food manufacture, consumers might also eat a part of visceral mass. *C. a. maximum* snail fillet weighted (2.1±2.1) g and *H. lucorum* (1.6±0.6) g. Only mass of farmed (1.1±0.4) g and wild (1.0±0.3) g *C.a. aspersum* snails were the same (p>0.05) (Table 1). In literature, there are studies about histology and proximate composition of snails, but mass of snail fillet has been assessed here for the first time.

### Microstructure profile of snail fillet

**Fig. 1:**
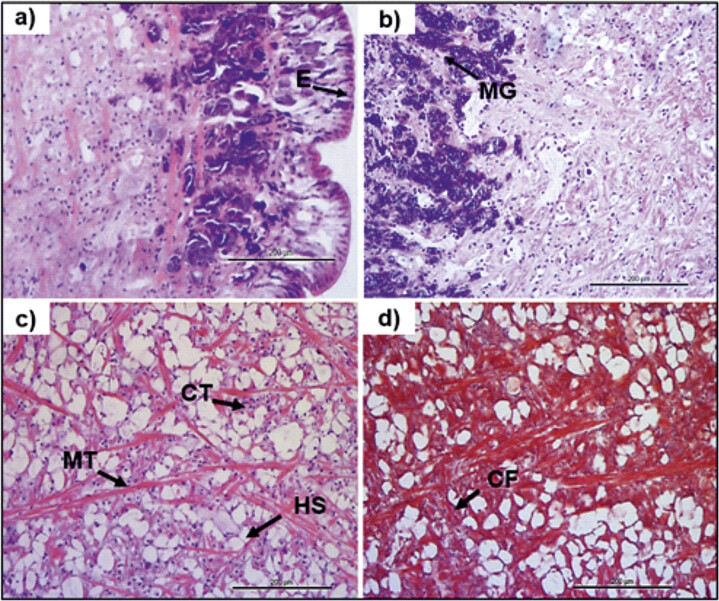
Micrographs of the mid-posterior region of raw fillet of *C. a. maximum* snail after: a−c) haematoxylin/eosin staining and d) Masson’s trichrome stain. For the analysis longitudinal (a, c, d) and transverse (b) sections were used. CF=collagen fibre, CT=connective tissue, E=epithelium, HS=system of haemocoelic sinuses (empty spaces), MT=muscle tissue, MG=mucus gland. Scale bar: 200 μm

The histological analysis was conducted on the cylindrical part (Fig. S1) of the middle-posterior region of fillet, which had specific shape and size, used for textural assessment. The suitability of middle-posterior region is explained by the absence of parts of digestive, reproductive and nervous systems, which are in the anterior region. Histological structure of the fillet of farmed *C. a. maximum* is illustrated in Fig. 1.

According to Fig. 1a, the integument in the ventral region had a flat rough surface and was covered by an epithelium with macroscopically visible infoldings. Ventral epithelium of the fillet was thicker than the dorsal region, as it was also mentioned in a recent study (*9*), according to which the ventral epithelium of the snail *Cepaea hortensis* was found to be twice thicker than the epithelium on the dorsal side.

Mucus glands were embedded in epithelium and subepithelial matrix of the fillet (Fig. 1b). Most of the secretory cells in pedal sole were of a distinct kind that produced mucus combined with protein (*10*, *11*).

Moreover, based on the histological analysis of *C. a. maximum* fillet, we identified the subepithelial matrix of muscle cells and connective tissue (Fig. 1c). The connective tissue was traversed by different types of cells such as rhogocytes, glycogen cells including packed glycogen and secretory cells which contain proteins, calcium, pigments, fat globules and mucus (*10*). In terms of muscle tissue, muscle cells showed different orientation and formed bunches (Fig. 1c). Czarnoleski *et al.* (*12*) studied the histological structure of farmed *C.a. aspersum* and *C.a. maximum* kept at 15 and 20 °C, and fed with food that included plants, minerals and vitamins, enriched with dry soil and an addition of CaCO_3_. Even though higher temperature led to smaller muscle and epithelial cells in both species, the overall conclusion of that study was that the farmed *C.a. aspersum* consisted of larger muscle and epithelial cells than the farmed *C.a. maximum*, regardless of breeding temperature (*12*).

The matrix of muscle and connective tissue was interwoven by numerous capillaries of the system of haemocoelic sinuses where haemolymph is gathered (Fig. 1c). Gastropods have open circulatory system and haemolymph circulates throughout the body and serves also as a hydroskeleton (*12*).

We indicated that muscle cells were surrounded by collagen fibre after Masson trichrome staining in sections of mid-posterior region of the fillet (Fig. 1d). Collagen fibre size might present difference between farmed and wild populations. According to Berillis *et al.* (*13*), collagen fibre of the farmed snails *C. a. aspersum* were bigger ((38.8±7.6) nm) than those of the wild snails of the same species ((32.7±7.2) nm).

### Compositional analysis of snail meat

According to Table 2, wild *C. a. aspersum* snails had the highest moisture mass fraction ((83.3±1.0) %) and farmed snails of the same species had the lowest mass fraction ((81.8±1.5) %). *C. a. maximum* snails had moisture mass fraction (83.0±1.6) % and *H. lucorum* snails (82.2±1.6) %. Based on our analysis, crude protein ranged from 10.3 to 13.5% and the wild species *H. lucorum* had a richer protein mass fraction ((13.5±0.1) %) than the farmed species *C. a. maximum* ((10.3±0.3) %). Farmed and wild snails *C. a. aspersum* had the same value (11.0%) (Table 2). Similarly, wild species *C.a. aspersum* (0.7±0.3) % and *H. lucorum* (0.6±0.1) % were found to have higher fat content than the farmed species *C.a. aspersum* ((0.4±0.1) %) and *C. a. maximum* ((0.1±0.0) %). As it is illustrated in Table 2, raw fillets of wild and farmed *C. a. aspersum* had the highest mass fraction (1.5%) of ash content, while *C. a. maximum* snails had the lowest ((1.1±0.0) %). *H. lucorum* snails had (1.3±0.1) % ash mass fraction. The energy content ranged from (20.0±0.2) to (21.1±0.2) kJ/g (Table 2). According to the results in the present study, even though *H. lucorum* snails had the highest energy content_,_ they had the lowest carbohydrate mass fraction ((2.4±0.1) %). Raw fillets of the other wild species, *C. a. aspersum,* contained (3.4±0.4) % and snails of the farmed species *C. a. maximum* and *C. a. aspersum* (5.5±0.2) and (5.4±0.5) % of carbohydrates, respectively.

**Table 2:** Proximate composition of different snail species fillets

Parameter	*C. a. maximum* (farmed)	*C. a. aspersum* (farmed)	*C. a. aspersum* (wild)	*H. lucorum* (wild)
*w*(moisture)/%	83.0±1.6	81.8±1.5	83.3±1.0	82.2±1.6
*w*(crude protein)/%	10.3±0.3	11.0±0.5	11.0±0.6	13.5±0.1
*w*(crude fat)/%	0.1±0.0	0.4±0.1	0.7±0.3	0.6±0.1
*w*(ash)/%	1.1±0.0	1.5±0.1	1.5±0.5	1.3±0.1
*E*/(kJ/g)	20.5±0.15	20.3±0.2	20.0±0.2	21.1±0.2
*w*(carbohydrate)/%	5.5±0.2	5.4±0.5	3.4±0.4	2.4±0.1

Results are expressed as mean value±S.D. (*N*=3)

Based on the results of our study, raw fillets of all the species had similar proximate composition to that reported in literature (*3*-*5*, *38*). Proximate composition of farmed snail species is affected by farming system and diet. According to Gomot (*4*), fillets of *C. a. maximum* snails which ate an artificial feed showed higher fat mass fraction (0.8%) than the fat mass fraction reported in this study (0.1%) in the fillets of snails of the same species that were supplied from a net-covered greenhouse in Central Greece. Milinsk *et al.* (*5*, *39*) reported that proximate composition of *C. a. maximum* snail body might change based on a diet. Even though the feed of the farmed *C. a. maximum* used in this study was supplemented with calcium, the ash mass fraction of their fillet was the lowest. Moreover, Gomot (*4*) found a slightly lower ash mass fraction of 1.3% in the fillet of *C. a. aspersum* snails fed with E3-2 than the ash mass fraction of 1.5% of our fillets from snails derived from an open field farm and fed with plants. On the contrary, the ash mass fraction of the *H. lucorum* snail fillets was the same (1.3%) in both studies. In the present study, artificially fed *C. a. maximum* snails had higher carbohydrate mass fraction (5.5%) than the mass fraction (2.0%) reported by Gomot (*4*).

### Hardness evaluation of snail fillets

Hardness is a texture-related property that describes resistance of a product to deformation or breaking (*40*). Many foods are processed and formulated with a large number of ingredients, but it is not difficult to control overall textural properties (*41*, *42*). On the other hand, there are foods with texture characteristics related to their original native microstructure, such as raw snails, of which, when processed to fillets, size is reduced and the hardness changes. There are also many factors that affect hardness of raw food, such as chemical composition, breeding and environmental factors, and usually there is no direct correlation between the composition and hardness (*40*). Hardness of snails was only assessed by Schubring and Meyer (*22*). The authors used whole body of snails for texture assessment and reported the highest hardness value of 50.2 N in *Achatina fulica*, then 27.4 and 23.5 N in *H. lucorum* and *Helix pomatia*, respectively, and the lowest value of 18.5 N in *C. a. aspersum*.

Due to the natural variations, shape and size of snail fillets cannot be completely identical. To eliminate these dissimilarities, we also deformed the cylindrical parts of the fillets to specific dimensions of 6 mm diameter and 6 mm height, except for hardness measurements, where we used the whole snail fillets. According to the histological analysis, the chosen part has a more uniform microstructure.

**Fig. 2:**
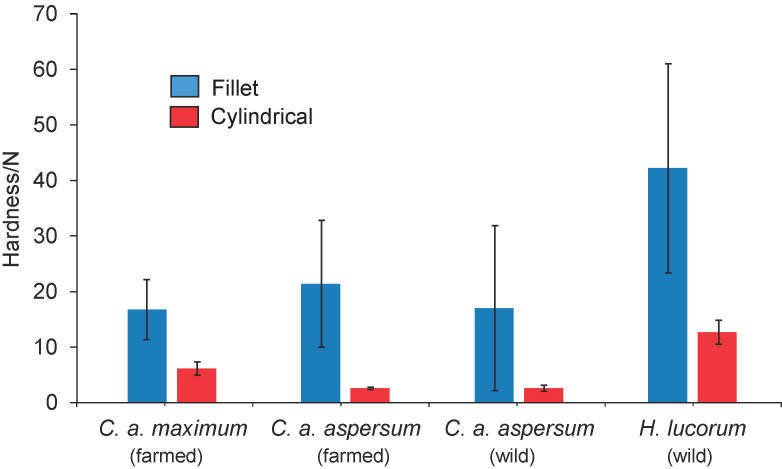
Hardness values (mean value±S.D.) of whole raw fillets and their cylindrical parts of farmed *C. a. maximum*, farmed and wild *C. a. aspersum and* wild *H. lucorum* snails

As the chosen cylindrical part is of a smaller size than the complete fillets, hardness values of the fillets were higher than those of cylindrical parts, (Fig. 2). *F*_max_ values of whole fillets ranged from 16.7 to 42.2 N, while the hardness of cylindrical parts ranged from 2.6 to 12.7 N. More specifically, hardness values of *H. lucorum* showed the highest value of 42.2 N among fillet groups and 12.7 N among cylindrical part groups. The fillet hardness of this species was higher than of *C. a. maximum* (16.7 N) and wild *C.a. aspersum*. Fillets of farmed *C. a. aspersum* were harder (21.4 N) than the fillets of wild *C. a. aspersum* (17.0 N) (Fig. 2). *C. a. maximum* cylindrical parts were not the softest among the groups (6.1 N). Based on our results, cylindrical parts of farmed and wild *C. a. aspersum* were softer and reported the same hardness value (2.6 N). The highest hardness values obtained for cylindrical parts were found for *H. lucorum* sample, which had the highest nitrogen content. We found the same results for whole fillet analysis. This is the first time that a part of snail fillet of specific size and shape has been used to evaluate hardness in order to minimize natural, breeding and environmental influences.

### Colour assessments of snail fillets

Colour measurements of each type of snail fillet are given in Table 3. Snail fillets were exposed to air at a chiller temperature (5 °C) before the surface of the ventral region colour was evaluated (*31*). The highest L* parameter (lightness) (49.2) was measured in farmed *C. a. aspersum*. *C. a. maximum* also had the highest a* (redness) and b* (yellowness) parameters (5.4 and 14.2, respectively). Snail fillets of species *C. a. maximum* were lighter (45.9) than wild *C. a. aspersum* (35.2), while the fillets of *H. lucorum* had the lowest mean value of L* (32.7). We reported almost the same value of a* (1.0 and 1.1 respectively) in the fillets of farmed and wild *C. a. aspersum*, while the wild snails of this species were more yellow (9.4) than the farmed ones (6.5). *H. lucorum* were redder (4.6) but less yellow (7.8) than the other wild species, *C. a. aspersum*. Differences in chroma are meaningful, but for hue big differences were not detected among snail samples. *C. a. maximum* has the highest (15.2) and farmed *C. a. aspersum* the lowest (6.6) chroma value. Wild species *C. a. aspersum* and *H. lucorum* have similar values of chroma.

**Table 3:** Colour parameters of different snail species fillets

Species	L*	a*	b*	*h* ^0^	C*
*C. a. maximum* (farmed)	45.9±3.1	5.4±0.7	14.2±2.1	1.2	15.2
*C. a. aspersum* (farmed)	49.2±3.1	1.0±0.2	6.5±0.8	1.4	6.6
*C. a. aspersum* (wild)	35.2±2.2	1.1±0.2	9.4±1.3	1.5	9.5
*H. lucorum* (wild)	32.7±4.2	4.6±1.3	7.8±1.6	1.0	9.1

Results are expressed as mean value±S.D (*N*=5). L*= lightness, a*=redness, b*=yellowness, *h*^0^=hue, C*=chroma

Today there are not many investigations of snail colour. For fresh *C. a. aspersum*, Cagiltay *et al.* (*3*) reported higher values of L* (54.7±1.8) and b* (19.5±1.5) and a* close to zero. Schubring and Meyer (*22*) reported colour parameters of treated minced snails. *C. a. aspersum* fillets were less bright (31.0) but the reddest (4.0) among the species. The values of L*, a* and b* for *H. lucorum* snails were 39.6, 2.3 and 7.4, respectively.

In our research colour parameters were studied for wild and farmed snail fillets. The species of farmed snails (*C. a. maximum* and *C. a. aspersum*) showed higher values of L* and higher carbohydrate mass fractions than the wild ones (*C. a. aspersum* and *Helix lucorum*). Although the farmed snails have lighter colour skin due to their feeding, L* parameter could also be related to their meat carbohydrate content as these species have higher carbohydrate mass fractions. *Helix lucorum* fillet skin is dark brown and it has the lowest carbohydrate mass fraction (2.4±0.1) and the lowest L* value (32.7±4.2). Differences observed in colour parameters b*, chroma and hue were less essential. The farmed species *C. a. maximum* was evaluated with the highest values of a*, b* and chroma compared to other species.

## CONCLUSIONS

Τhis study gives qualitative characteristics and important information on chemical composition, hardness and colour properties of wild and farmed snail fillets. This is the first time that hardness of a part of specific size and shape of snail fillet has been evaluated to minimize the influences such as age, diet, farming and/or environmental conditions. Histological analysis supports and describes the uniform structure of the specific part of snail fillet. The chemical analysis of *Helix lucorum* snail fillet showed the highest energy content and the highest hardness, but the lowest carbohydrate content. Farmed snail fillets provide significantly higher brightness and had higher carbohydrate content than the wild species. Finally, the quality characteristics of wild and farmed snail fillets and the novel hardness determination method could give information for further processing of snail meat in food industry.
